# Predicting mostly disordered proteins by using structure-unknown protein data

**DOI:** 10.1186/1471-2105-8-78

**Published:** 2007-03-06

**Authors:** Kana Shimizu, Yoichi Muraoka, Shuichi Hirose, Kentaro Tomii, Tamotsu Noguchi

**Affiliations:** 1 Department of Computer Science, Graduate School of Science and Engineering, Waseda University, 3-4-1 Okubo, Shinjuku-ku, Tokyo 169-8555, Japan; 2 Computational Biology Research Center (CBRC), National Institute of Advanced Industrial Science and Technology (AIST), 2-42 Aomi, Koto-ku, Tokyo 135-0064, Japan; 3 PharmaDesign, Inc, 2-19-8 Hatchobori, Chuo-ku, Tokyo 104-0032, Japan

## Abstract

**Background:**

Predicting intrinsically disordered proteins is important in structural biology because they are thought to carry out various cellular functions even though they have no stable three-dimensional structure. We know the structures of far more ordered proteins than disordered proteins. The structural distribution of proteins in nature can therefore be inferred to differ from that of proteins whose structures have been determined experimentally. We know many more protein sequences than we do protein structures, and many of the known sequences can be expected to be those of disordered proteins. Thus it would be efficient to use the information of structure-unknown proteins in order to avoid training data sparseness. We propose a novel method for predicting which proteins are mostly disordered by using spectral graph transducer and training with a huge amount of structure-unknown sequences as well as structure-known sequences.

**Results:**

When the proposed method was evaluated on data that included 82 disordered proteins and 526 ordered proteins, its sensitivity was 0.723 and its specificity was 0.977. It resulted in a Matthews correlation coefficient 0.202 points higher than that obtained using FoldIndex, 0.221 points higher than that obtained using the method based on plotting hydrophobicity against the number of contacts and 0.07 points higher than that obtained using support vector machines (SVMs). To examine robustness against training data sparseness, we investigated the correlation between two results obtained when the method was trained on different datasets and tested on the same dataset. The correlation coefficient for the proposed method is 0.14 higher than that for the method using SVMs. When the proposed SGT-based method was compared with four per-residue predictors (VL3, GlobPlot, DISOPRED2 and IUPred (long)), its sensitivity was 0.834 for disordered proteins, which is 0.052–0.523 higher than that of the per-residue predictors, and its specificity was 0.991 for ordered proteins, which is 0.036–0.153 higher than that of the per-residue predictors. The proposed method was also evaluated on data that included 417 partially disordered proteins. It predicted the frequency of disordered proteins to be 1.95% for the proteins with 5%–10% disordered sequences, 1.46% for the proteins with 10%–20% disordered sequences and 16.57% for proteins with 20%–40% disordered sequences.

**Conclusion:**

The proposed method, which utilizes the information of structure-unknown data, predicts disordered proteins more accurately than other methods and is less affected by training data sparseness.

## Background

Various kingdoms of life appear to have proteins or protein segments that lack a folded structure [1-3]. These proteins and segments are thought to be intrinsically disordered structures providing essential biological functions [4-8], so predicting such disorder should help us understand protein functions. Disorder have been found in proteins involved in regulatory and signaling events [4,9-11] and may provide conformational flexibility that allows proteins to interact with several structurally different targets [5,12,13].

Many studies have shown that the primary structure of disordered regions is distinct from that of structured regions [14], and this has encouraged the development of many prediction methods based on the amino acid sequence. PONDR [15], GlobPlot [16], DISOPRED [17,18], VL3 [19], DISEMBL [20], IUPred [21], and RONN [22] predict the probability of any given residue being in a disordered region by using information about the amino acid sequences near that residue. A different approach predicts disorder by binary classification of amino acid sequences into mostly disordered sequences and mostly ordered sequences [23-25]. The former approach is based on the view that features of the local sequence are a more important than features of the whole structure.

Two methods have been used for binary classification. Uversky et al. suggested that a mostly disordered protein sequence could be discriminated from an ordered one by plotting the average hydrophobicity of the residues in the sequence against the net charge of the sequence [23,24], and that method has been implemented as the web-based FoldIndex application [26]. Garbuzynskiy et al., on the other hand, classified proteins as ordered or disordered by estimating the number of contacts of the whole protein [25]. Both methods classify a target protein by using a linear discriminant function.

Linear discriminant analysis, like other classification methods, infers a discriminant function that minimizes the misclassification of training data. The parameter optimization of the linear equation is therefore strongly influenced by the distribution of training data. In the prediction of protein disorder, the analysis depends on the protein sequences that are already known to be folded or unfolded. The training will be successful if the amount of training data is large enough to approximate the distribution of all protein sequences. If the quantity of training data is too small, however, the classification boundary overfits to a local cluster of protein structures.

It is hard to find disordered proteins not only because protein structures are often determined by X-ray diffraction analysis and information about proteins that could not be crystallized for X-ray analysis is seldom reported but also because not every failure to crystallize is due to disorder. Previous studies have estimated the proportion of disordered proteins in various genomes. PONDR estimated that 60% of eukaryotic proteins and 28% of bacterial proteins include disordered regions more than 40 residues long [1,27]. DISOPRED2, on the other hand, estimated that 33.0% of eukaryotic proteins and 4.2% of bacterial proteins include disordered regions longer than 30 residues and estimated that no more than 0.5% of the sequences in the Protein Data Bank (PDB) include disorder regions longer than 30 residues [2]. Despite the appreciable frequency of disordered proteins, the sequences of few mostly disordered proteins are publicly available. Although the current version (release 3.3) of DisProt [28], which is a public database providing information about disordered proteins, provides the sequences of 458 proteins, only 82 of those proteins are more than 70% disordered.

We therefore think that protein databases might be biased against disordered proteins. If there are a lot of unknown disordered proteins, the structural distribution of proteins in nature will differ from that of proteins whose structure has been determined experimentally. Since training classifiers on data biased in this respect neglects of the actual distribution of natural proteins, the discriminative boundary should be adjusted to compensate the sparseness of the training data. Semi-supervised learning has been gaining increasing attention for dealing with problems due to data sparseness. Conventional supervised-learning methods, including support vector machines (SVMs) and neural networks, use only labeled data when optimizing the parameters of the discriminant function. In binary classification, the labeled data is a set of samples each of which is known to be positive or negative. When semi-supervised learning builds a model to improve predictions, it takes into account not only the labeled data but also the unlabeled data by adapting to the distribution of unlabeled data.

A huge amount of known-sequence data is available. UniProt (UniProt50 release 48.9), for example, which is a widely used database of protein sequences, contains 974,638 nonredundant proteins [29], many of which can be expected to include a lot of disorder. We therefore, think it efficient to utilize the information of structure-unknown proteins by using semi-supervised learning to avoid training data sparseness. And prediction that considers a robust model will provide a new indicator for protein disorder.

In this study we developed a novel method for predicting disordered proteins by using Joachims' spectral graph transducer (SGT) [30], which is a binary classification algorithm based on semi-supervised learning. It constructs a k-nearest neighbor (kNN) graph with both labeled and unlabeled examples as vertices, and the edge weight between two vertices represents their similarity. If the graph is separated into two subgraphs, both labeled and unlabeled vertices are classified into two categories. The SGT takes into account both the prediction accuracy of labeled training data and the distribution of unlabeled data, because it cuts the kNN graph so as to minimize both the misclassification of labeled vertices and the sum of edges weights across the cut. We apply the SGT to the disorder prediction problem with structure-known sequences as labeled data and structure-unknown sequences, including query sequences, as unlabeled data. The proposed method can therefore be used for training both structure-known sequences and a huge amount of structure-unknown sequences, and it creates a model that incorporates a larger protein structural space. We examined how data with no structural information improves the prediction of disordered proteins and we compared the accuracy of the proposed SGT-based method with the accuracy of an SVMs-based method and the accuracies of two other previous methods. We compared this SGT-based binary-classification method with per-residue methods by comparing their predictions for both mostly disordered proteins and mostly ordered proteins. We also estimated the false positive rate when the proposed method was used for partially disordered proteins.

## Results and Discussion

### Effect of structure-unknown proteins on disorder prediction

Since the SGT constructs a model on both labeled data and unlabeled data, the accuracy of its predictions is influenced structure-unknown sequences as well as structure-known sequences. Here we examine how structure-unknown sequences affect prediction accuracy.

#### Does structure-unknown data increase prediction accuracy?

We tested different quantities of unlabeled samples in order to find out whether structure-unknown sequences have a positive or negative effect.

The SGT classifies unlabeled data as either a disordered protein or an ordered protein, so query sequences are also treated as unlabeled data. We tried to increase prediction accuracy by using as unlabeled data not only query sequences but also large numbers of structure-unknown protein sequences.

**Figure 1:**
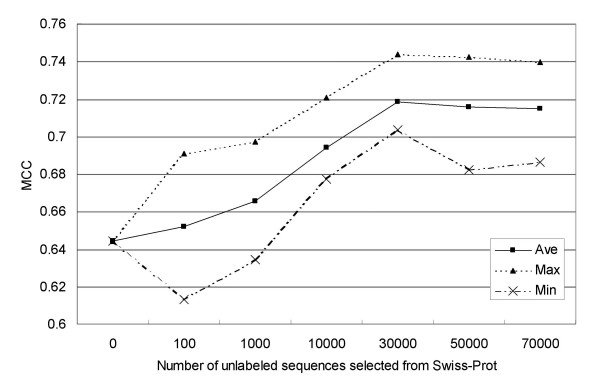
Average (square), maximum (triangle) and minimum (X) values of Matthews correlation coefficient (MCC) for 10 different datasets in each of the seven experiments with different quantities of unlabeled data.

To investigate the effect of structure-unknown sequences, we prepared different quantities of unlabeled samples that were added to query sequences. Each set of unlabeled samples (structure-unknown sequences) was chosen randomly from the Swiss-Prot database. We prepared 10 different datasets for each experiment in order to avoid sampling bias, and the results we obtained are shown in Figure 1. Note that the x-axis in Figure 1 does not include the number of query sequences, and the total number of unlabeled samples in these experiments was the sum of the number of query sequences and the number of proteins selected from the Swiss-Prot database.

As shown in Figure 1, the maximum, minimum and average Matthews correlation coefficient (MCC) for the 10 datasets were highest in the experiment with 30,000 structure-unknown samples selected from the Swiss-Prot database. The computational cost of building and decomposing a kNN graph increases with the number of examples, so smaller numbers of examples are more practical with respect to computation time. Since the average MCC is almost the same for 30,000, 50,000 and 70,000 examples, 30,000 is the most practical number of examples as well as the one yielding the highest average MCC.

The recent research reported that regions of predicted disorder were found to be conserved within a large number of protein families and domains [31]. The proposed method considers information about conserved regions through similarities among sequences. The results shown in Figure 1 indicate that the proposed method effetely utilized information about conservation of protein disorder.

#### Curated data or uncurated data?

To investigate whether classification accuracy is affected by the quality of unlabeled samples, we used unlabeled samples from three different databases: Swiss-Prot, UniProt50 and TrEMBL. Each set of 30,000 or 70,000 unlabeled samples was chosen randomly, and we used 10 datasets from each database in order to avoid sampling bias. The results are listed in Table 1. Swiss-Prot outperformed UniProt and TrEMBL, which indicates the quality of unlabeled data is an important factor for prediction accuracy.

**Table 1:** Average sensitivity (SEN), specificity (SPC), two-state accuracy (Q2) and Matthews correlation coefficient (MCC) for 10 different data sets in each experiment with different databases.

	SEN	SPC	Q2	MCC
30000 unlabeled sequences				
Swiss-Prot	0.684	0.978	0.938	0.718
Uniprot	0.581	0.976	0.922	0.636
TrEMBLE	0.567	0.980	0.925	0.643

70000 unlabeled sequences				
Swiss-Prot	0.701	0.973	0.936	0.715
Uniprot	0.570	0.979	0.923	0.638
TrEMBLE	0.504	0.978	0.914	0.585

Swiss-Prot is a reliable database, which is carefully organized by human curators. TrEMBL is a computer-annotated supplement to Swiss-Prot, which contains amino acid translations of all the EMBL nucleotide sequence entries, including sequences automatically predicted by gene-finding programs. UniProt consists of Swiss-Prot and TrEMBL. This means that TrEMBL and UniProt might include a lot of artificial translation of pseudogenes, which are not translated into proteins *in vivo*. Such databases containing noise sequences are inferred to have a background distribution distinct from that of native protein sequences, and this distinction would have a negative effect on prediction.

#### Which similarity measurement is best?

SGT divides a kNN graph into two subgraphs for binary classification. Since the edge weight of the graph represents the similarity of two vertices, a similarity measurement for two protein sequences has to be defined. We based predictions on three measurements (amino acid composition, composition of physicochemical properties and BLAST score) and examined which is best. The results obtained using 30,000 structure-unknown sequences are listed in Table 2. The amino acid composition yielded the best results (most discriminative predictions), and the BLAST score yielded the worst results (least discriminative predictions). Compositionally based similarity measurements were thus better for predicting dissimilar proteins than was motif or sequence similarity measurement.

**Table 2:** Average sensitivity (SEN), specificity (SPC), two-state accuracy (Q2) and Matthews correlation coefficient (MCC) for predictions using different similarity measurements: amino acid composition (AA comp), physicochemical property composition (PP comp) and BLAST score.

	SEN	SPC	Q2	MCC
AA Comp	0.684	0.978	0.938	0.718
PP Comp	0.569	0.982	0.926	0.650
BLAST Score	0.663	0.920	0.885	0.546

### Comparison with previous methods

**Figure 2:**
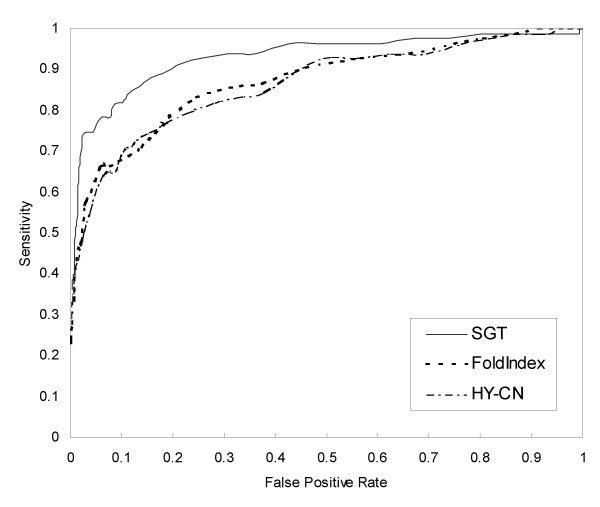
ROC curves comparing our SGT-based method and previous methods.

We compared the proposed method with two previous methods: FoldIndex [23] and plotting hydrophobicity against the number of contacts (hydrophobicity-contactnumber plot) [25]. FoldIndex output was obtained from the web server provided by the Israel Structural Proteomics Center, and we implemented a method for hydrophobicity-contactnumber plot because no web server or tool was available. We optimized its parameters on the same data we used for training our SGT-based method. For the proposed method, amino acid composition similarity measurement and 30,000 structure-unknown samples were used. The results are listed in Table 3. The proposed method yielded a MCC 0.202 points greater than that obtained using FoldIndex and yielded a MCC 0.221 points greater than that obtained using the method calculating the number of hydrophobic residues in contact. We also show Receiver Operating Characteristic (ROC) curves in Figure 2.

**Table 3:** Sensitivity (SEN), specificity (SPC), two-state accuracy (Q2) and Matthews correlation coefficient (MCC) for the proposed method (SGT), FoldIndex, the method plotting hydrophobicity against the number of contacts (HY-CN) and an SVMs-based method (SVMs).

	SEN	SPC	Q2	MCC
SGT	0.723	0.977	0.943	0.744
FoldIndex	0.663	0.918	0.884	0.542
HY-CN	0.663	0.909	0.876	0.523
SVMs	0.614	0.979	0.930	0.673

**Figure 3:**
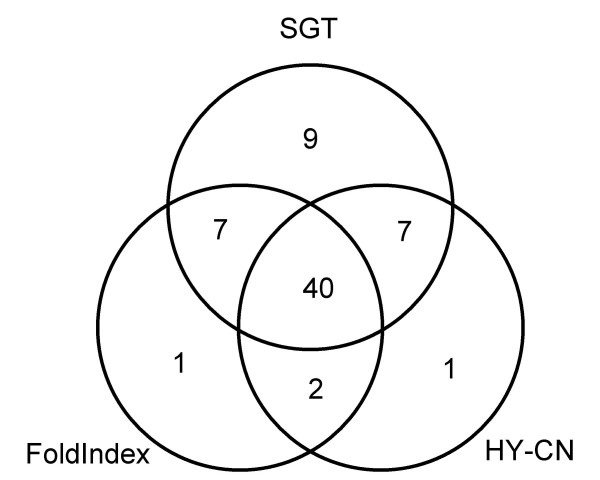
Comparing true positives among the SGT-based method, FoldIndex and the method plotting hydrophobicity against the number of contacts (HY-CN).

The proposed method has two advantages over previous methods. The first advantage is that it can construct a nonlinear classification boundary taking account of the background distribution of a large amount of protein sequences, which enables the classifier to avoid training data sparseness. The second advantage is that it uses more information than the previous methods do. FoldIndex and hydrophobicity-contactnumber plot postulate that a few physicochemical properties of target proteins are the main factor of disorder. Although this strategy provides a simple and clear indicator, it overlooks many disordered proteins because other complex factors are involved in protein disorder. Classification over a larger feature space should facilitate more accurate prediction. Simple indicators cannot express some features of disorder. Plotting average hydrophobicity against net charge or the number of residues in contact, for example, does not always reflect the sequence complexity, which is an important factor in the discrimination of disordered proteins [32]. If an amino acid that is used repeatedly has average hydrophobicity, net charge and contact number values, such information will remain hidden. Although the composition of the entire sequence cannot be used to distinguish the local sequence complexity, it reflects long-range complexities. We conjecture that the hydropobicity-vs-(net charge) and hydropobicity-vs-(contact number) feature spaces should be considered subsets of the amino-acid-composition feature space. The two advantages of the proposed method enable it to identify more disordered proteins. For the same false positive rate (5%), SGT found 23 disordered proteins that FoldIndex or plotting hydrophobicity against contact number did not find and found nine proteins that neither previous method found. And neither FoldIndex nor plotting hydrophobicity against contact number found four disordered proteins that SGT did not find (Figure 3).

### Comparison with support vector machines

Many forms of biological data are classified using support vector machines, neural networks, or other types of traditional machine learning. These algorithms are supervised learning procedures where the classifier is trained on labeled data. Spectral Graph Transducer, which is associated with semi-supervised learning, differs from other forms of supervised learning in that it uses unlabeled data. We also tested SVMs with the same features we use in SGT in order to investigate whether semi-supervised learning with unlabeled sequences is effective for predicting disorder. We compared SGT with SVMs, which is known to be a powerful classifier and has been widely applied to biological data analyses, using amino acid compositions as the feature vector. The SVMs package libSVM was used; Performance of major three kernels (linear, polynomial, RBF) was compared, and RBF kernel, which gave the best result, was used. All parameters were tuned by grid-search. For SGT, 30,000 structure-unknown samples were used. These results are also shown in Table 3. SGT gave a MCC 0.07 points better than the SVMs did.

Supervised learning methods, including SVMs, are especially sensitive to the training data distribution when the given data set is a small one. Therefore, if biased data are provided, the predictive tendency will differ even if predictions are made using the same data. We compared the predictive tendencies with different training data as follows: (1) evaluation data were divided into three groups (Data-A, Data-B and Data-C); (2) each datum was predicted using a classifier trained on different data; and (3) the correlation coefficient between the two results was calculated. (e.g., a classifier trained on Data-A classifies all sequences from Data-C, another classifier trained on Data-B also classifies all sequences of Data-C. and then the coefficient of the correlation between two results is calculated). The average correlation coefficient for SGT was 0.14 higher than that for the SVMs (Table 4). An SGT-based method, which uses a huge number of unlabeled samples, makes prediction robust with regard to training data sparseness. This result indicates that SGT prediction is less affected by training data bias and provides accurate predictions even with a poor data set. Experimentally determined protein structures can potentially bias the data set. Previous research has shown that discriminating disorder from order is similar to finding the classification boundary between crystal structures and solution structures [14]. This is an unavoidable problem as long as a limited dataset is used, but distribution of structure-unknown data modified the training data bias.

**Table 4:** Correlation coefficients for the proposed method (SGT) and an SVMs-based method (SVMs) trained on different datasets.

	Data-A	Data-B	Data-C	Average
SGT	0.92	0.85	0.94	0.90
SVMs	0.83	0.83	0.63	0.76

### Comparison with per-residue predictors

There are many studies in which the probability of any given residue being in a disordered region was predicted. Although the methods used in those studies are not directly comparable to our method, comparing the proposed method to per-residue predictors gives helpful information about the accuracy of the proposed method.

**Figure 4:**
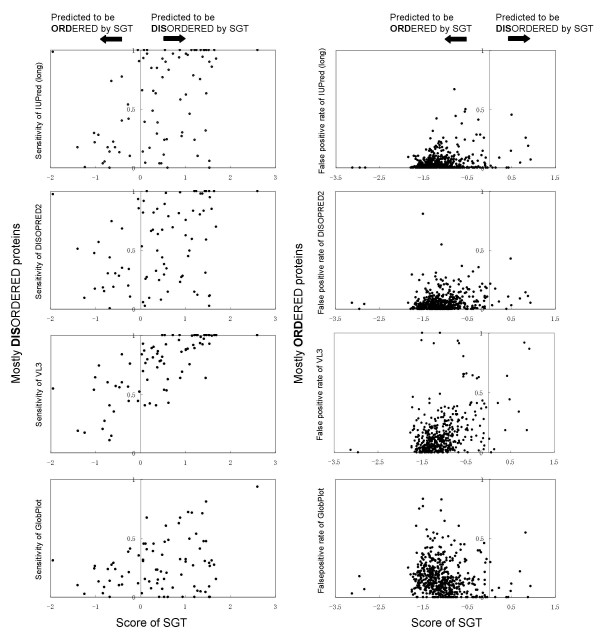
Relations between the SGT score and the sensitivities and false positive rates of four per-residue predictors.

We select four successful per-residue predictors for comparison: VL3 [19], GlobPlot [16], DISOPRED2 [18] and IUPred (long) [21]. The VL3, GlobPlot and IUPred (long) results were obtained from web servers, and the DISOPRED2 results were obtained from a stand-alone program [33]. Detailed results are shown in Figure 4, which shows the results of two types of evaluation. In the graphs on the left side, showing results for mostly disordered proteins (at least 70% of their residues are disordered), the sensitivities of each per-residue predictor are plotted against the SGT scores. In the graphs on the right side, showing results for mostly ordered proteins (at least 95% of their residues are ordered), the false positive rates of each per-residue predictor are plotted against the SGT scores. The SGT gives each protein a score that shows how likely the protein is to be disordered. It assigns positive score when it predicts a query protein to be disordered. For example, if a point is plotted in the lower right portion of one of the graphs on the left, the proposed method can correctly classify the target sequence while the corresponding per-residue predictor cannot find a lot of disordered residues.

Table 5 also compares per-residue sensitivity on mostly disordered proteins, sensitivity and specificity on mostly ordered proteins of the proposed method to those of per-residue predictors. When we evaluated SGT prediction, we regarded all residues of the target protein to be predicted to be disordered if the SGT assigned positive score to the protein. And we also regarded all residues of the target protein to be predicted to be ordered if the SGT assigned a negative score to the protein. (I.e., the sensitivity of the proposed method becomes 0.7 if the SGT score is positive and the query sequence includes 70 disordered residues and 30 ordered residues).

**Table 5:** Per-residue sensitivity (SEN) on mostly disordered proteins (average disorder length is 228.54) and sensitivity (SEN) and specificity (SPC) on mostly ordered proteins (average disorder length = 2.47) for the proposed method (SGT) and four per-residue predictors.

	Mostly DISORDERED proteins Ave DR length = 228.54	Mostly ORDERED proteins Ave DR length = 2.47	
	SEN	SEN	SPC

SGT	0.834	0.009	0.991
VL3	0.782	0.562	0.880
IUPred (long)	0.666	0.262	0.955
Disopred2	0.645	0.780	0.954
GlobPlot	0.311	0.324	0.838

Specificity for mostly disordered proteins cannot be calculated because sequences selected from DisProt have no information for ordered regions. The 71 predictions by VL3 for mostly ordered sequences were excluded because the VL3 server does not return results for sequences that include ambiguous characters such as 'Z'.

DISOPRED2 and IUPred have low false positive rates on mostly ordered proteins. DISOPRED2 successfully predicts short disordered regions in mostly ordered proteins (proteins with an average disorder length of 2.47 residues per sequence), but does not detect 35.5% of the disordered regions in mostly disordered proteins (proteins with an average disorder length of 228.54 residues per sequence). VL3, on the other hand, successfully finds 78.2% of the disordered regions in mostly disordered proteins but produces a lot of false positives on mostly ordered proteins.

These per-residue predictors try to find exact position of disorder by classifying a fixed window length to be disordered or ordered. Because their prediction thus concentrates on the local trend of disorder, they miss the global trend of disorder. And because a shorter window size includes less information, the local trend of disorder is more difficult to discriminate than the global trend of disorder. When a classification scheme such as neural networks or SVMs is used to determine a classification boundary between similar examples, there is inevitably a trade-off between getting a large number of false positives and getting a large number of true positives. This trade-off is strongly influenced by ratio of positive/negative training examples.

Predicting disorder by classifying proteins as mostly disordered or mostly ordered is a rough approximation but has the advantage of detecting long disordered regions with a low false positive rate by neglecting short disordered regions. The proposed method predicts 83.4% of the disordered regions in mostly disordered proteins, and its false positive rate on mostly ordered proteins is only 0.9%. We therefore think insight is obtained by predict both disordered regions and disordered proteins, since a region-based-prediction provides information local trends of disorder while a protein-based-prediction gives information about large-area trends of disorder.

### Evaluation on partially disordered proteins

Here we describe the results of prediction on partially disordered proteins. Not all proteins are mostly disordered or mostly ordered and many are partially disordered. Evaluating our method on partially disordered proteins gave us practical information we could use for estimating the false positive rates that would occur when it is used for large-scale genome analysis. As shown in Table 6, the proposed method is insensitive for the partially (5–20%) disordered proteins, although the method can predict that 16.67% of the moderately (20–40%) disordered proteins are disordered.

**Table 6:** Frequency of partially disordered proteins predicted to be mostly disordered by the proposed method.

Disorder frequency f	Number of proteins predicted to be disordered	Total number of proteins	frequency of proteins predicted to be disordered (%)
5% ≤ f < 10%	5	256	1.95
10% ≤ f < 20%	2	137	1.46
20% ≤ f < 50%	4	24	16.67

### Prediction of disordered proteins in large databases

To provide illustrative examples of novel predictions made by our SGT-based method, we made predictions on several databases.

Ward et al., using DISOPRED2, estimated 18.9% of eukaryotic genomes and 5.7% of bacterial genomes to be disordered and found long (> 30 residues) disordered segments in 2.0% of archaean proteins, 4.2% of bacterial proteins and 33.0% of eukaryotic proteins [2]. Bogatyreva et al., evaluating the expected number of contacts, estimated that 12%, 3% and 2% of the proteins in eukaryotic, bacterial and archaean proteomes are totally disordered and that long (> 41 residues) disordered segments occur in 16% of archaean proteins, 20% of bacterial proteins and 43% of eukaryotic proteins [34]. The proposed method predicts that an average of 4.14% of archaean proteins, 7.0% of bacterial proteins and 28.5% of eukaryotic proteins are mostly disordered. The frequencies estimated for 5 archaean, 14 bacterial and 5 eukaryotic genomes, in addition to the overall totals for each domain, are listed in Table 7. In line with the results of previous genome-wide analysis, eukaryotic genomes are predicted to code for much more disorder than prokaryotic genomes do. This is consistent with much experimental evidence that has shown that dynamic flexibility of the protein structure is more often related to eukaryotic protein function than to bacterial and archaean protein function [12].

**Table 7:** Estimated frequencies of disordered protein in 24 representative genomes.

Kingdom	Species	Number of total sequences	Disordered protein frequency (%)
Archaea	Halobacterium sp. NRC-1	2605	4.57
Archaea	Pyrococcus horikoshii	1535	2.41
Archaea	Thermoplasma volcanium	1526	3.87
Archaea	Sulfolobus solfataricus	2977	3.76
Archaea	Nanoarchaeum equitans	536	9.89
Bacteria	Escherichia coli K-12	4302	4.21
Bacteria	Acidobacteria bacterium Ellin345	4777	4.92
Bacteria	Staphylococcus aureus RF122	2515	5.81
Bacteria	Mycobacterium tuberculosis H37Rv	3991	4.03
Bacteria	Fusobacterium nucleatum	2067	5.22
Bacteria	Rhodopirellula baltica	7325	14.06
Bacteria	Chlamydophila pneumoniae AR39	1110	9.91
Bacteria	Treponema pallidum T. pallidum	1031	6.89
Bacteria	Synechocystis sp. PCC6803	3454	5.07
Bacteria	Porphyromonas gingivalis	1909	7.70
Bacteria	Chlorobium tepidum C. tepidum	2255	7.54
Bacteria	Dehalococcoides ethenogenes	1580	6.39
Bacteria	Deinococcus radiodurans	3181	3.99
Bacteria	Thermotoga maritima	1846	7.04
Eukaryota	Arabidopsis thaliana	25545	22.51
Eukaryota	Caenorhabditis elegans	22844	21.33
Eukaryota	Drosophila melanogaster	19376	30.21
Eukaryota	Homo sapiens	40877	36.85
Eukaryota	Saccharomyces cerevisiae	5869	18.73

Archaea		9179	4.14
Bacteria		41343	7.00
Eukaryota		114511	28.50

**Figure 5:**
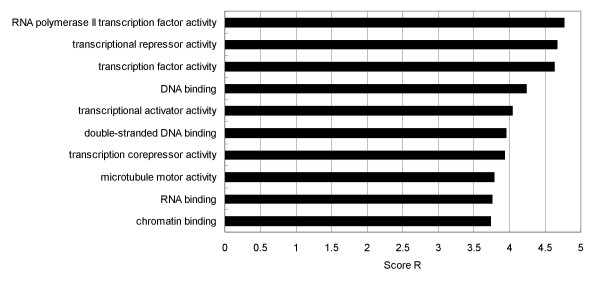
The 10 GO molecular function terms with the highest normalized ratios R.

**Figure 6:**
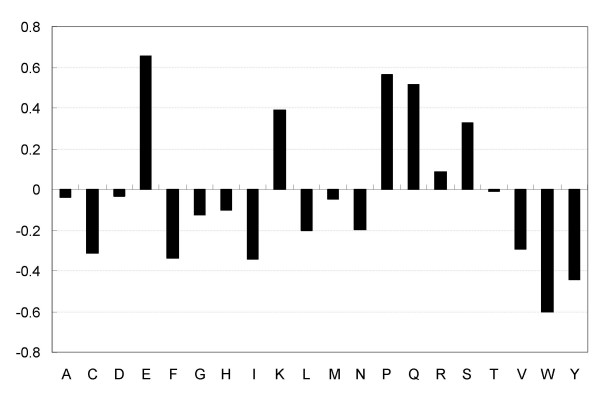
**Disorder propensities of 20 amino acids**. Each propensity was calculated as *D*_*i*_/*O*_*i*_, where *D*_*i *_is the frequency of amino acid *A*_*i *_in disordered proteins and *O*_*i *_is the frequency of amino acid *A*_*i *_in ordered proteins.

**Figure 7:**
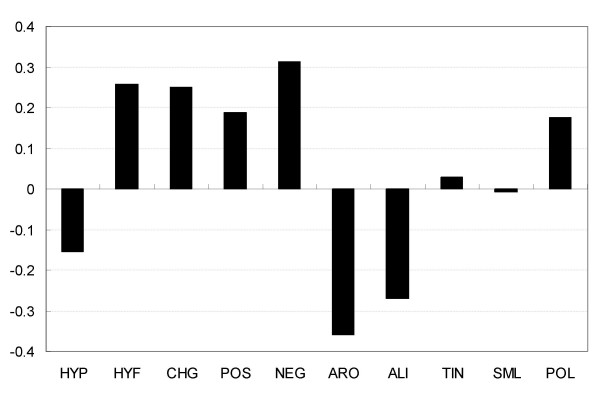
**Disorder propensities of 10 physicochemical properties**. Each propensity was calculated as *D*_*i*_/*O*_*i*_, where *D*_*i *_is the frequency of amino acids with the physicochemical feature *P*_*i *_in disordered proteins and *O*_*i *_is the frequency of amino acids with physicochemical feature *P*_*i *_in ordered proteins. (HYP: hydrophobic, HYF: hydrophilic, CHG: charged, POS: positively charged, NEG: negatively charged, ARO: aromatic, ALI: aliphatic, TIN: tiny, SML: small, POL: polar)

The proposed method also predicts that 15.46% of all sequences in the Swiss-Prot database are disordered. To investigate functional annotations of those sequences that were predicted to be disordered, we calculated the normalized ratio of annotated GO molecular function terms: *R*(*T*) = *R*_*d*_(*T*)/*R*_*s*_(*T*), where *R*_*d*_(*T*) is the ratio of the proteins annotated by GO term *T *to all the proteins predicted to be disordered and *R*_*s*_(*T*) is the ratio of the proteins annotated by GO term *T *to all the sequences in the Swiss-Prot database. The top 10 of the GO molecular function terms that describe more than 50 protein annotations, the 10 with the highest normalized ratios *R*_*d*_(*T*) are listed in Figure 5. The proposed method was biased to find transcriptional-factor-related, RNA-binding-related and DNA-binding-related proteins to be disordered. Binding to nucleic acids requires interaction between the nucleic acid phosphate backbone and charged amino acids, which have a propensity for disorder (disorder propensities of amino acids and physiochemical properties are shown in Figures 6 and 7). Therefore it is not necessarily appropriate to suggest that all RNA-binding and DNA-binding proteins need dynamic flexibility, though previous papers have discussed the relation between disorder and proteins binding RNA and DNA [2,10,31,35]. The global analysis over large databases by the proposed method is an on-going study, and in the future we will use the proposed method to find new disordered proteins and will promote its use in further functional analysis of disordered proteins in collaboration with experimental laboratories.

## Conclusion

In this study we proposed a semi-supervised learning approach for predicting disordered proteins. Disordered proteins are getting more and more attention because many of them are found to be functionally important. Few proteins however, are known to be disordered because information about a protein that could not be crystallized for X-ray analysis is seldom reported, even if the protein might be disordered. We therefore expect the distribution of disorder among proteins whose structure has been determined experimentally to differ from that of disorder among all natural proteins. Since the predictions made by previous methods are based on structure-known data, they are strongly affected by the bias for information about readily crystallized proteins. To avoid training data sparseness and to structure the hypothesis space based on the entire protein distribution, we have proposed a prediction method that uses Joachims' spectral graph transducer and is trained on both structure-known sequences and structure-unknown sequences.

This method yielded MCCs 0.202 points higher than the MCC yielded by the method plotting hydrophobicity vs. net charge (FoldIndex) and 0.221 points higher than the MCC yielded by the method plotting hydrophobicity against the number of contacts. When the false positive rate was 5%, we found 23 disordered proteins that were not found using those previous methods.

The proposed method predicts disorder by classifying proteins as either mostly disordered or mostly ordered. While such binary classification cannot detect partially disordered regions that per-residue predictors can find, it has the advantage of detecting long disordered regions by neglecting short disordered regions. When the proposed SGT-based method was compared with four per-residue predictors-VL3, GlobPlot, DISOPRED2, IUPred (long)-its sensitivity for disordered proteins was 0.834, which is 0.052–0.523 higher than that of the per-residue predictors and its specificity for ordered proteins was 0.991, which is 0.036–0.153 higher than that of the per-residue predictors. 

The main contribution of this paper is that it provides a method in which structure-unknown protein sequences are used to increase the accuracy with which disordered proteins can be predicted. We compared the results obtained using the proposed method with the results obtained using a SVMs-based method that used the same features that the proposed method used (the composition of 20 amino acids). The proposed method resulted in a MCC 0.07 points higher than the MCC obtained using the SVMs-based method. When it and the method using SVMs were trained on two different datasets and both methods were tested on a third dataset, it provided an average correlation coefficient that was 0.14 higher than that provided by the method using SVMs. The SGT-based prediction was less affected by training data sparseness and provided more accurate predictions when the data set was a poor one. These results provide convincing evidence for a positive effect of structure-unknown protein sequences, and our SGT-based method is therefore able to serve as a new indicator of disordered protein that considers the overall protein distribution in nature.

## Methods

### Materials

#### Disordered proteins

We downloaded the current version of DisProt (version 3.3) and extracted proteins having more than 70% disorder. Then we clustered those sequences by sequence similarity of 30% using BASTclust, and selected representative sequences. We thereby obtained 82 sequences.

#### Ordered proteins

The data was prepared according to the following protocol. Complex proteins were excluded because their folded regions are possible to be unfolded on a single state. Because X-ray crystallographic analysis induces artifactually missing residues, high-quality data and well-refined data were selected in steps (2) and (3). BLASTclust was used for task (6).

1. Extract single-chain proteins from Protein Data Bank.

2. Extract data that has a resolution better than 2 Å and an observed R-factor less than 0.2.

3. Extract data determined by a newer version than Refmac5, SHELXL97 or CNS.

4. Extract proteins that are more than 95% ordered.

5. Exclude proteins that show disorder in the central area (between the 10th residue from the N-terminal end and the 10th residue from the C-terminal end).

6. Choose a representative sequence with 30% similarity to avoid redundancy.

We thereby obtained 526 sequences.

#### Unlabeled proteins

We used Uniprot50 (downloaded on 12 Jan 2006: 974,638 sequences), Swiss-Prot (release 48.9: 206,586 sequences), and TrEMBL (downloaded on 2 Feb 2006: 2,586,884 sequences). Short sequences tend to have a biased amino acid composition, which adversely affects prediction. We therefore excluded sequences shorter than 30 residues when it is used for semi-supervised training of SGT.

#### Partially disordered proteins

the data were prepared according to the following protocol. Complex proteins were excluded because their folded regions are possible to be unfolded on a single state. Because X-ray crystallographic analysis induces artifactually missing residues, high-quality data and well-refined data were selected in steps (2) and (3).

1. Extract single-chain proteins from Protein Data Bank.

2. Extract data that has a resolution better than 2 Å and an observed R-factor less than 0.2.

3. Extract data determined by a newer version than Refmac5, SHELXL97 or CNS.

4. Extract proteins that include more than 5% disorder.

We thereby obtained 417 sequences.

#### Protein sequences of 24 genomes

We used proteins sequences for 5 archaean, 14 bacterial and 5 eukaryotic genomes that were downloaded on 18 August 2006 from the NCBI ftp server.

### Spectral Graph Transducer

The spectral graph transducer (SGT) is a powerful binary classification algorithm that was developed by Joachims [30]. It is based on semi-supervised learning, which for training makes use of not only labeled data (for which the answer is known) but also unlabeled data (for which the answer is unknown). This type of learning method often improves the prediction accuracy obtained when only a small amount of labeled data is available.

A goal of the classifier is to assign a label (either +1 or -1) to unlabeled examples. The SGT takes into account the information of unlabeled data by using a graph composed of both labeled data and unlabeled data. Given a set of labeled examples *L *= *l*_0_,...,*l*_*m *_and unlabeled examples *U *= *u*_0_,...,*u*_*n*_, the SGT constructs a k-nearest-neighbor graph *G *with *X *= {*U*, *L*} as vertices. The graph *G *has *n *+ *m *vertices, and edge weights between the vertices represent the similarity of the neighboring examples. The SGT assigns a label (either +1 or -1) to *U *by dividing *G *into two subgraphs *G*^+ ^and *G*^- ^(∀*u*_*i *_∈ *G*^+ ^are assigned +1, ∀*u*_*i *_∈ *G*^- ^are assigned -1) That is, *G*^+ ^and *G *^- ^define a cut in the graph. The SGT chooses the cut so that it provides a small training error (i.e., an *l*_*i *_that is labeled +1 should belong to *G*^+^), has a low cut cost (i.e., it minimizes the sum of the edge weights across the cut) and makes the ratio of positive examples to negative examples in *U *the same as it is in *L*. This strategy is implemented by minimizing:

miny→y→T(B−A)y→+c(y→−γ→)TC(y→−γ→)
 MathType@MTEF@5@5@+=feaafiart1ev1aaatCvAUfKttLearuWrP9MDH5MBPbIqV92AaeXatLxBI9gBaebbnrfifHhDYfgasaacH8akY=wiFfYdH8Gipec8Eeeu0xXdbba9frFj0=OqFfea0dXdd9vqai=hGuQ8kuc9pgc9s8qqaq=dirpe0xb9q8qiLsFr0=vr0=vr0dc8meaabaqaciaacaGaaeqabaqabeGadaaakeaafaqabeqacaaabaacbiGae8xBa0Mae8xAaKMae8NBa42aaSbaaSqaaiqbdMha5zaalaaabeaaaOqaaiqbdMha5zaalaWaaWbaaSqabeaacqWGubavaaGccqGGOaakcqWGcbGqcqGHsislcqWGbbqqcqGGPaqkcuWG5bqEgaWcaiabgUcaRiabdogaJjabcIcaOiqbdMha5zaalaGaeyOeI0ccciGaf43SdCMbaSaacqGGPaqkdaahaaWcbeqaaiabdsfaubaakiabdoeadjabcIcaOiqbdMha5zaalaGaeyOeI0Iaf43SdCMbaSaacqGGPaqkaaaaaa@4C49@

s.t.y→T1=0andy→Ty→=n+m,
 MathType@MTEF@5@5@+=feaafiart1ev1aaatCvAUfKttLearuWrP9MDH5MBPbIqV92AaeXatLxBI9gBaebbnrfifHhDYfgasaacH8akY=wiFfYdH8Gipec8Eeeu0xXdbba9frFj0=OqFfea0dXdd9vqai=hGuQ8kuc9pgc9s8qqaq=dirpe0xb9q8qiLsFr0=vr0=vr0dc8meaabaqaciaacaGaaeqabaqabeGadaaakeaafaqabeqaeaaaaeaacqqGZbWCcqGGUaGlcqqG0baDcqGGUaGlaeaacuWG5bqEgaWcamaaCaaaleqabaGaemivaqfaaOGaeGymaeJaeyypa0JaeGimaadabaGaeeyyaeMaeeOBa4MaeeizaqgabaGafmyEaKNbaSaadaahaaWcbeqaaiabdsfaubaakiqbdMha5zaalaGaeyypa0JaemOBa4Maey4kaSIaemyBa0MaeiilaWcaaaaa@4547@

where

Aij=wij∑k∈kNN(xi)wik,Bii=∑jAijBij=0(i≠j)
 MathType@MTEF@5@5@+=feaafiart1ev1aaatCvAUfKttLearuWrP9MDH5MBPbIqV92AaeXatLxBI9gBaebbnrfifHhDYfgasaacH8akY=wiFfYdH8Gipec8Eeeu0xXdbba9frFj0=OqFfea0dXdd9vqai=hGuQ8kuc9pgc9s8qqaq=dirpe0xb9q8qiLsFr0=vr0=vr0dc8meaabaqaciaacaGaaeqabaqabeGadaaakeaafaqabeqadaaabaGaemyqae0aaSbaaSqaaiabdMgaPjabdQgaQbqabaGccqGH9aqpdaWcaaqaaiabdEha3naaBaaaleaacqWGPbqAcqWGQbGAaeqaaaGcbaWaaabeaeaacqWG3bWDdaWgaaWcbaGaemyAaKMaem4AaSgabeaaaeaacqWGRbWAcqGHiiIZcqqGRbWAcqqGobGtcqqGobGtcqGGOaakcqWG4baEdaWgaaadbaGaemyAaKgabeaaliabcMcaPaqab0GaeyyeIuoaaaGccqGGSaalaeaacqWGcbGqdaWgaaWcbaGaemyAaKMaemyAaKgabeaakiabg2da9maaqafabaGaemyqae0aaSbaaSqaaiabdMgaPjabdQgaQbqabaaabaGaemOAaOgabeqdcqGHris5aaGcbaGaemOqai0aaSbaaSqaaiabdMgaPjabdQgaQbqabaGccqGH9aqpcqaIWaamcqGGOaakcqWGPbqAcqGHGjsUcqWGQbGAcqGGPaqkaaaaaa@611F@

and *y*_*i *_is the prediction score of *x*_*i*_. If *y*_*i *_> 0, +1 is assigned to *x*_*i*_. The term *γ*_*i *_is the penalty if *x*_*i *_∈ *L *is misclassified. Therefore *γ*_*i *_is positive for *x*_*i *_∈ *L*^+^, negative for *x*_*i *_∈ *L*^- ^and 0 for *x*_*i *_∈ *U*. The *c *is a parameter that trades off training error against cut cost, and *C *is a diagonal cost matrix that allows different misclassification costs for each example.

The spectral graph transducer outperforms other semi-supervised learning methods in many benchmark datasets [30]. We used the SGT package SGT*light *in our experiments. Since classification accuracy is little affected by changing the two parameters *c *(trade-off of wrongly classifying training data) and *d *(number of eigenvectors) [30], we used *c *= 10,000 and *d *= 100. For the number of nearest neighbors, we tried *k *= 80, 90,...,120 and selected the one giving the best results (*k *= 100).

### Similarity of two sequences

Because SGT is a kNN-based algorithm, the similarity of two sequences must be defined. We used three types of similarity measurement:

#### 1. Amino acid composition

The amino acid composition is a basic property of proteins. Figure 6 shows the disorder propensities of 20 amino acids. A vector with 20 elements for the amino acid composition was used to calculate cosign.

#### 2. Composition of physicochemical properties

Previous work has shown that composition alone is sufficient to recognize disorder accurately. Even a reduced alphabet of amino acids is useful for accurate prediction [36]. Figure 7 shows propensity for disorder of 10 physicochemical properties. We used a vector having 10 elements for physicochemical properties to calculate cosign. The binary definition of the physicochemical features is according to Zvelebil et al. [37].

#### 3. Sequence similarity

Both of the two similarity measurements described above are based on compositional biases of amino acids. We also proposed a measurement based on sequence similarity or local motif. Top k raw score of BLAST search are used as similarity score between query sequence and database sequences for constructing kNN graph. The database of the BLAST search consists of training sequences.

### Evaluation

We used five-fold cross validation for our experimental evaluation as follows.

1. We separate evaluation data (608 sequences) into five data sets and selected one (e.g., 121 or 122 sequences) for test data. The rest of the data (486 or 487 sequences) was used as training data.

2. Labels of test data are hidden.

3. Construct k-NN graph using training data, test data and proteins which are selected from Swiss-Prot.

4. Separate the k-NN graph into two (disordered or ordered) for prediction. Each sequence of unlabeld data (test data and proteins which are selected from Swiss-Prot) was classified as disordered or ordered.

5. We evaluate the precision of test data.

Steps 1–5 were repeated five times with different training data and test data.

Sensitivity (*tp*/(*tp *+ *fn*)), specificity (*tn*/(*tn *+ *fp*)), two-state accuracy ((*tp *+ *tn*)/(*tp *+ *tn *+ *fp *+ *fn*)), false positive rate (*fp*/(*tn *+ *fp*)) and the Matthews correlation coefficient (MCC) were used for the evaluation. Because sensitivity and specificity are trade-off criteria, we needed a balancing criterion for the MCC. This criterion was calculated as

(tn∗tp)−(fn∗fp)(tp+fp)∗(tn+fn)∗(tp+fn)∗(tn+fp),
 MathType@MTEF@5@5@+=feaafiart1ev1aaatCvAUfKttLearuWrP9MDH5MBPbIqV92AaeXatLxBI9gBaebbnrfifHhDYfgasaacH8akY=wiFfYdH8Gipec8Eeeu0xXdbba9frFj0=OqFfea0dXdd9vqai=hGuQ8kuc9pgc9s8qqaq=dirpe0xb9q8qiLsFr0=vr0=vr0dc8meaabaqaciaacaGaaeqabaqabeGadaaakeaadaWcaaqaaiabcIcaOiabdsha0jabd6gaUjabgEHiQiabdsha0jabdchaWjabcMcaPiabgkHiTiabcIcaOiabdAgaMjabd6gaUjabgEHiQiabdAgaMjabdchaWjabcMcaPaqaamaakaaabaGaeiikaGIaemiDaqNaemiCaaNaey4kaSIaemOzayMaemiCaaNaeiykaKIaey4fIOIaeiikaGIaemiDaqNaemOBa4Maey4kaSIaemOzayMaemOBa4MaeiykaKIaey4fIOIaeiikaGIaemiDaqNaemiCaaNaey4kaSIaemOzayMaemOBa4MaeiykaKIaey4fIOIaeiikaGIaemiDaqNaemOBa4Maey4kaSIaemOzayMaemiCaaNaeiykaKcaleqaaaaakiabcYcaSaaa@6285@

where *tp *is the number of true positives, *tn *the number of true negatives, *fp *the number of false positives and *fn *the number of false negatives.

## Availability

**Project Name: **POODLE-W

**Project Home Page: **

**Operating Systems: **POODLE-W is a web application that can be accessed from any OS.

**Programming languages: **C++, Perl(for CGI programming).

**Restrictions to use by non-academics: **none.

## Authors' contributions

KS designed the methodology, developed the programs, implemented the experiments and did most of the writing under the guide of YM. SH and KT provided helpful insight in experiment and discussion. TN initiated the project. All authors contributed to the final version of the manuscript and approved it.
